# Score-matched analysis of *human papillomavirus* photodynamic therapy with *Nocardia rubra* and aminolevulinic powder

**DOI:** 10.1097/MD.0000000000046369

**Published:** 2026-05-12

**Authors:** Suzhen Jiang, Jincheng Huang, Xiaomei Nan, Miaoxia Huang, Hongxia Gong

**Affiliations:** a Dongguan Songshan Lake Tungwah Hospital, Dongguan, China; b Dongguan SongShan Lake Central Hospital, Dongguan, China.

**Keywords:** aminolevulinic acid hydrochloride topical powder photodynamic therapy, high-grade squamous intraepithelial lesion, high-risk *human papillomavirus*, low-grade squamous intraepithelial lesion, *Nocardia rubra* cell wall skeleton, thinprep cytologic test

## Abstract

Effective clearance of high-risk *human papillomavirus* (HR-HPV) and disease remission are critical in managing cervical lesions. This study compared *Nocardia rubra* cell wall skeleton (Nr-CWS) versus 5-aminolevulinic acid photodynamic therapy for HR-HPV clearance and lesion regression. In this single-center retrospective cohort (June 2020–May 2023), 234 patients with HR-HPV infection were assessed. After 1:1 propensity matching, 144 patients remained (72 per group). Primary and secondary endpoints were HR-HPV clearance and disease remission rates, respectively. Baseline differences in thinprep cytologic test results persisted post-matching. Nr-CWS demonstrated superior HR-HPV clearance at 6 months (76.4% vs 48.6%; *P* < .001) and 1 year (87.5% vs 66.7%; *P* = .03). Disease remission rates showed no significant differences at 6 months (72.9% vs 85.5%; *P* = .10) or 1 year (78.0% vs 90.9%; *P* = .058). Nr-CWS significantly outperformed 5-aminolevulinic acid photodynamic therapy in achieving HR-HPV clearance, while both therapies showed comparable efficacy in disease remission.

## 1. Introduction

Cervical cancer is overwhelmingly linked to high-risk *human papillomavirus* (HR-HPV) infection, more than 99.7% of cases attributable to persistent HR-HPV.^[1]^ HR-HPV16 and HR-HPV18 are the predominant oncogenic strains globally; however, in China, HR-HPV52, HR-HPV58, and HR-HPV33 also contribute significantly to invasive cervical cancer and precancerous lesions (e.g., low-grade squamous intraepithelial lesion [LSIL]/cervical intraepithelial neoplasia [CIN] I).^[2,3]^ A large-scale analysis of 444,471 participants revealed a 10.8% HR-HPV prevalence among Chinese women aged 35 to 64.^[4]^ Persistent infection not only heightens cancer risk but also imposes psychological distress and facilitates transmission to sexual partners.^[5]^ Critically, residual HR-HPV infection after conization surgery (e.g., LEEP/CKC) for high-grade squamous intraepithelial lesion markedly increases recurrence risk, underscoring the need for effective interventions.^[6]^

Topical immune modulators like *Nocardia rubra* cell wall skeleton (Nr-CWS) enhance cervical immunity, promoting HR-HPV clearance and demonstrating efficacy in treating persistent HR-HPV with LSIL.^[7,8]^ Similarly, 5-aminolevulinic acid photodynamic therapy (ALA-PDT) effectively targets HR-HPV-infected lesions and intraepithelial abnormalities.^[9–11]^ Recent recommendations outline the appropriate use, treatment protocols, procedural steps, and safety measures for administering ALA-PDT in the management of lower female reproductive tract diseases.^[12]^ Both Nr-CWS and ALA-PDT represent promising noninvasive strategies for HR-HPV clearance.^[13–15]^ Nevertheless, a critical knowledge gap remains: no studies have directly compared the long-term efficacy of Nr-CWS versus ALA-PDT for HR-HPV using rigorous methods like propensity score matching (PSM).

This study aims to compare HR-HPV clearance rates between Nr-CWS and ALA-PDT therapies, with secondary endpoints assessing disease remission rates (e.g., regression of LSIL). Our propensity score-matched analysis will evaluate long-term outcomes to inform optimized clinical management of persistent HR-HPV infection.

## 2. Materials and methods

### 2.1. Data collection

We retrospectively reviewed records of HR-HPV patients undergoing treatment at the Department of Dongguan SongShan Lake Tungwah Hospital, Guangdong Province, China between June 2020 and May 2023. Exclusion criteria were as follows: Non-exposure to Nr-CWS or ALA-PDT; Treatment interruption/modality change; Lost to follow-up; Lesion progression during follow-up. The patients were divided into 2 groups: Nr-CWS and aminolevulinic acid hydrochloride topical powder photodynamic. Collected variables: Age, gravidity, parity, delivery mode, HR-HPV type, thinprep cytologic test (TCT), biopsy pathology, vaginal cleanliness, cervical transformation zone, contraception, menopause status. This article describes a study that was ruled exempt from institutional review board approval because it used data gathered by observing public activity without interfering.

### 2.2. Photodynamic therapy

ALA-PDT was administered using 20% 5-aminolevulinic acid hydrochloride topical powder (Shanghai Fudan-Zhangjiang Bio-Pharmaceutical Co., Ltd., China) with an LED-IB photodynamic laser (Wuhan Ya Daylight Electric Technology Co., Ltd.). The procedure involved applying 1.5 mL of thermogel containing 20% ALA dispersant to the cervix, sealing it for 4 hours, followed by 633 nm laser irradiation at 80 J/cm² for 30 minutes. This treatment was repeated weekly for a total of 3 sessions. Patients selected their treatment option before enrollment and were monitored for adverse reactions during the treatment.

### 2.3. Nr-CWS for external use

Patients received Nr-CWS treatment from Liaoning Grist Biopharmaceutical Co., Ltd, dosed at 60 μg per branch, twice a day with a day in between, completing 10 applications over approximately 1 month. Before application, the vulva, vagina, and cervix were cleansed with normal saline, and the cervical surface was lightly abraded until minor bleeding occurred. A 1 mL syringe was used to scratch the cervix to a depth of 2 to 3 mm. The first dose was dissolved in 1 mL normal saline and administered into the cervical canal with a depth limit of 1 cm, ensuring the canal was filled without overflow. The second dose was applied using a cotton ball soaked in 2 mL normal saline, placed in contact with the cervix or lesion site, and left in place for 15 to 20 minutes before being removed 24 hours later.

### 2.4. Follow-up

Efficacy of Nr-CWS and ALA-PDT was evaluated at 6 and 12 months using HR-HPV DNA testing and TCT. For patients who did not complete tests at our hospital, we followed up via telephone or WeChat to obtain their results.

### 2.5. Statistical analysis

*Propensity score matching:* Variables included: Age, gravidity, parity, delivery mode, HR-HPV type, TCT, biopsy pathology, vaginal cleanliness, cervical transformation, contraception, menopause. Matching method: 1:1 greedy nearest-neighbor (caliper = 0.02). Outcome: 72 matched pairs.^[16]^

*Statistical methods:* Continuous data: Paired *t* test (mean ± SD). Categorical data: McNemar or *χ*^2^ test (n, %).

*Significance threshold: P* < .05 (2-tailed). Statistical analysis was performed with SPSS software (IBM Corp. Released 2020. IBM SPSS Statistics for Windows, Version 27.0. Armonk, NY: IBM Corp).

## 3. Results

**Figure 1.**
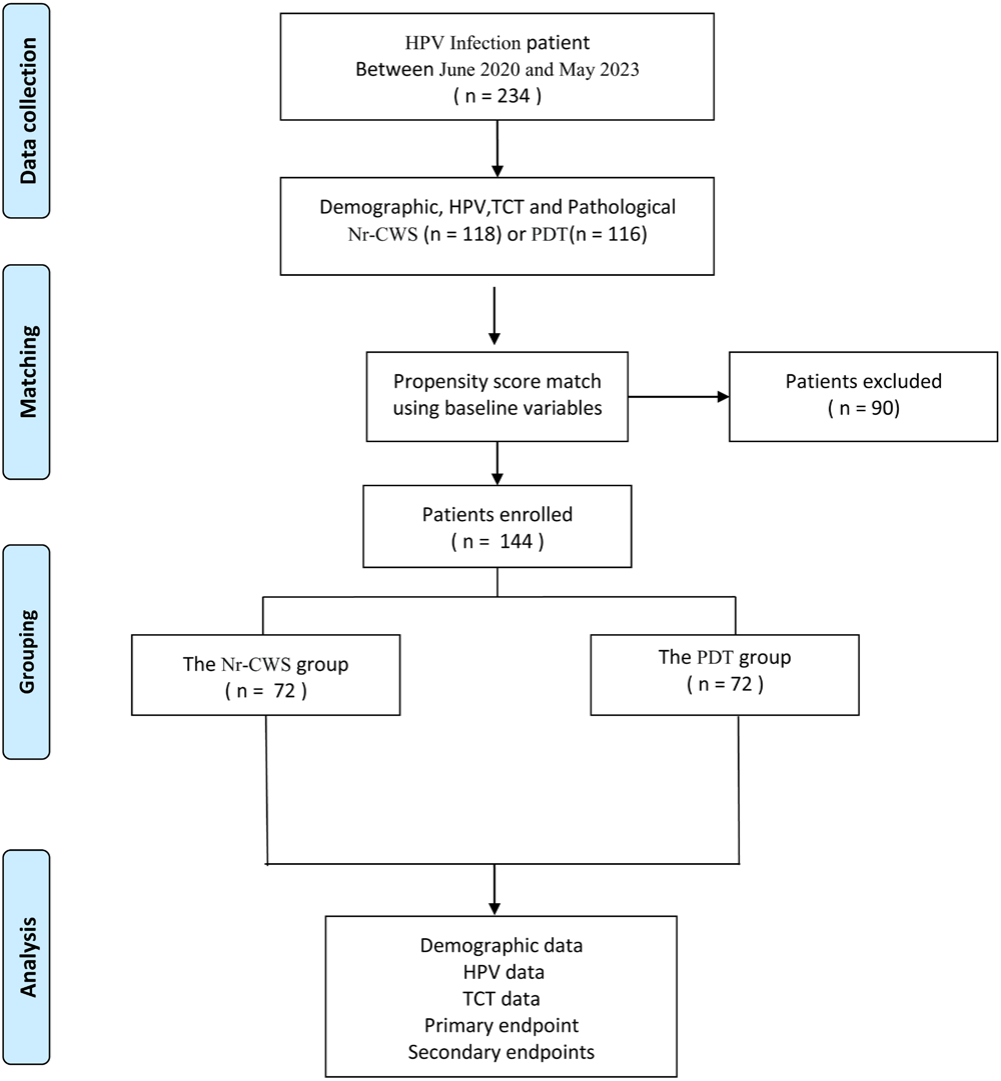
Flowchart illustrating patient selection and group allocation for a comparative study of HPV infection patients.

A total of 234 patients were included. A total of 142 eligible cases of HR-HPV infection were enrolled. After 1:1 PSM, 72 patients treated with Nr-CWS were compared to 72 patients receiving PDT (Fig. 1).

Baseline characteristics are summarized in Table 1. Significant differences existed pre-PSM in TCT, biopsy pathology, contraceptive methods, and menopausal status (*P* < .05). After PSM, all variables were balanced (*P* > .05) except TCT (*P* < .05).

**Table 1:** Baseline characteristics and distribution of cases (Nr-CWS and PDT) before and after propensity score matching.

	Total population			Propensity score-matched pairs		
	Nr-CWS (n = 118)	PDT (n = 116)	*P* value	Nr-CWS (n = 72)	PDT (n = 72)	*P* value
Age (y)	36.29 ± 9.01	37.4 ± 11.56	.396	35.89 ± 9.82	36.76 ± 10.74	.563
Number of pregnancies	3 (2–4)	3 (2–4)	.9	3 (2–4)	3 (2–4)	.887
Number of deliveries	2 (1.5–2.5)	2 (1.5–2.5)	.646	2 (1–2)	2 (1–2)	.963
Mode of delivery						
Vaginal delivery	67 (56.8)	69 (59.5)	.277	40 (55.6)	44 (61.1)	.409
Cesarean section	26 (22.0)	15 (12.9)		17 (23.6)	10 (13.9)	
Vaginal delivery and Cesarean section	6 (5.1)	7 (6.0)		2 (2.8)	4 (5.6)	
No delivery	19 (16.1)	25 (21.6)		13 (18.1)	14 (19.4)	
HPV infection						
16.18	50 (42.4)	38 (32.8)	.129	25 (34.7)	25 (34.7)	1.0
Non-16/18	68 (57.6)	78 (67.2)		47 (65.3)	47 (65.3)	
Single infection	73 (61.9)	79 (68.1)	.317	47 (65.3)	46 (63.9)	1.0
Multiple infections	45 (38.1)	37 (31.9)		25 (34.7)	26 (36.1)	
TCT						
Normal	73 (61.9)	41 (35.3)	<.001	45 (62.5)	32 (44.4)	.024
LSIL	20 (16.9)	48 (41.4)		10 (13.9)	27 (37.5)	
ASCUS	24 (20.3)	21 (18.1)		16 (22.2)	11 (15.3)	
Condyloma	1 (0.8)	6 (5.2)		1 (1.4)	2 (2.8)	
Biopsy pathology						
Normal	31 (26.3)	19 (16.4)	<.001	13 (18.1)	17 (23.6)	.373
Condyloma	4 (3.4)	13 (11.2)		3 (4.2)	8 (11.1)	
LSIL	81 (68.6)	69 (59.5)		54 (75)	46 (63.9)	
HSIL	2 (1.7)	15 (12.7)		2 (2.8)	1 (1.4)	
Vaginal discharge cleanliness						
II	35 (29.6)	22 (18.9)	.199	35 (29.6)	22 (18.9)	.518
III	68 (57.6)	81 (69.8)		68 (57.6)	81 (69.8)	
IV	15 (12.7)	13 (11.2)		15 (12.7)	13 (11.2)	
Cervical transformation zone						
I	27 (22.9)	19 (16.3)	.398	16 (22.2)	15 (20.8)	.935
II	29 (24.5)	24 (20.6)		16 (22.2)	17 (23.6)	
III	60 (50.8)	67 (57.7)		38 (52.8)	38 (52.8)	
Vaginal stump	2 (1.7)	6 (5.1)		2 (1.7)	2 (2.8)	
Contraceptive methods						
Condoms	41 (34.7)	62 (53.4)	.012	32 (44.4)	33 (45.8)	.690
Intrauterine device	7 (5.9)	7 (6.0)		7 (9.7)	4 (5.6)	
Contraceptive pills or no contraception	70 (59.3)	47 (40.5)		33 (45.8)	35 (48.6)	
Menopause						
Yes	8 (6.8)	20 (17.2)	.013	7 (9.7)	9 (12.5)	.846
No	108 (91.5)	90 (77.6)		63 (87.5)	61 (84.7)	
No uterus	2 (1.7)	6 (5.2)		2 (2.8)	2 (2.8)	

ASCUS = atypical squamous cells of undetermined significance, HPV = *human papillomavirus*, HSIL = high-grade squamous intraepithelial lesion, LSIL = low-grade squamous intraepithelial lesion, Nr-CWS = *Nocardia rubra* cell wall skeleton, PDT = photodynamic therapy, TCT = thinprep cytologic test.

Key treatment outcomes are detailed in Table 2 and demonstrated: Overall HR-HPV clearance: At 6 months, Nr-CWS achieved significantly higher clearance than PDT (76.4% vs 48.6%; *P* < .001). At 12 months, Nr-CWS maintained superior clearance (87.5% vs 66.7%; *P* = .03). No significant difference was observed in women without cervical lesions (e.g., 84.6% vs 58.8% at 6 months, *P* = .229; 84.6% vs 76.5% at 12 months, *P* = .672).

**Table 2:** HPV and TCT result after 6 and 12 months.

	6 months				12 months			
	Nr-CWS (n = 72)	PDT (n = 72)	*χ* ^2^	*P*	Nr-CWS (n = 72)	PDT (n = 72)	*χ* ^2^	*P*
HPV (-)	55 (76.4)	35 (48.6)	11.852	<.001	63 (87.5)	48 (66.7)	8.845	.03
TCT (-)	43 (72.9)	47 (85.5)	2.707	.1	46 (78.0)	50 (90.9)	3.586	.058
HPV① (-)	11 (84.6)	10 (58.8)	2.334	.229	11 (84.6)	13 (76.5)	0.305	.672
16, 18 (-)	19 (76.0)	10 (40.0)	6.65	.01	20 (80)	15 (66)	2.381	.123
Non-16/18 (-)	36 (76.6)	25 (53.2)	5.65	.017	43 (91.5)	33 (70.2)	6.871	.009
Multiple infections (-)	16 (64.0)	13 (50)	1.018	.313	21 (84.0)	17 (65.4)	2.325	.127
Single infection (-)	39 (83.0)	22 (47.8)	12.728	<.001	42 (89.4)	31 (67.4)	6.648	.01

HPV = *human papillomavirus*, HPV① = *human papillomavirus* without lesions, Nr-CWS = *Nocardia rubra* cell wall skeleton, PDT = photodynamic therapy, TCT = thinprep cytologic test.

*Clearance by HPV subtype:* HPV16/18: Significantly higher clearance with Nr-CWS at 6 months (76.0% vs 40.0%; *P* = .01), but no difference at 12 months (80.0% vs 66.0%; *P* = .123). Non-HPV16/18: Significantly higher clearance with Nr-CWS at both 6 months (76.6% vs 53.2%; *P* = .017) and 12 months (91.5% vs 70.2%; *P* = .009).

*Clearance by infection type:* Single infections: Nr-CWS yielded significantly higher clearance at 6 months (83.0% vs 47.8%; *P* < .001) and 12 months (89.4% vs 67.3%; *P* = .01). Multiple infections: No significant difference between groups at 6 months (64.0% vs 50.0%; *P* = .313) or 12 months (84.0% vs 65.4%; *P* = .127).

*Disease remission rate:* No statistically significant difference was observed between groups at 6 months (72.9% vs 85.5%; *P* = .100) or 12 months (78.0% vs 90.9%; *P* = .058).

## 4. Discussion

Comprehensive analyses report high remission rates for CIN and HR-HPV-associated lesions treated with PDT, reaching 81.0% (range: 31.3%–100%) and 80.4% (range: 53.4%–94.4%), respectively.^[17]^ HR-HPV clearance after PDT increases over time: 56.1% at 3 months and 68.1% at 6 months, with higher clearance observed in patients without cervical lesions.^[13]^

In our propensity score-matched cohorts comparing Nr-CWS and ALA-PDT for HR-HPV infection: PDT outcomes: Overall HR-HPV clearance ranged from 48.6% to 66.7%, rising from 58% (6 months) to 76.5% (12 months) in lesion-free patients. Cytology normalization was 85.5% (6 months) and 90.9% (12 months). Comparison: Our 6-month HR-HPV clearance was lower than some studies, but cytology normalization was higher. This may reflect fewer PDT sessions (3 vs typical 6), and the presence of cervical lesions in most patients may also affect the HR-HPV clearance rate.

Earlier research confirms PDT efficacy for CIN II/III (53.4%) HR-HPV clearance,^[18]^ noting that age and HR-HPV subtype influence ALA-PDT outcomes.^[13,19]^ Our data align, showing higher clearance for non-HPV16/18 subtypes (70.2%) versus HPV16/18 (66%).

Additionally, local treatment reactions, such as erythema, edema, pruritus, burning sensation, and pain, commonly occur in patients during and after ALA-PDT. These are considered common adverse reactions. According to a meta-analysis, the rate of PDT adverse events is 74.6%, while a systematic review reports a range of 31.6% (from 4.2% to 79.7%). Local reactions such as erythema and pain occur in 31.6% to 74.6% of ALA-PDT cases.^[12,17]^ Occasionally requiring discontinuation.

In contrast, Nr-CWS avoids these effects and achieves cytology normalization in 72.9%–78% of patients—comparable to PDT (85.5%–90.5%, *P* < .005). Due to potential side effects, treatment may need to be discontinued. Nr-CWS is an effective treatment for squamous intraepithelial lesions, capable of reducing lesion size and eliminating HR-HPV infections.^[20]^ Unlike PDT, Nr-CWS avoids treatment-related adverse effects. Literature reports its cytology normalization rate (87.8%) exceeds spontaneous remission and LEEP outcomes.^[21–23]^ Our propensity score-matched analysis shows that Nr-CWS achieves cytology normalization rates of 72.9%–78%, comparable to PDT (85.5%–90.5%; *P* < .005). At 6 months, the HR-HPV clearance rate for Nr-CWS was significantly higher than that for PDT (76.4% vs 48.6%; *χ*^2^ = 11.852, *P* < .001), and this trend continued at 12 months (87.5% vs 66.7%; *χ*^2^ = 8.845, *P* = .03). For single infections, the clearance rates were 83.0% (39/47) at 6 months and 89.4% (42/47) at 12 months, both significantly higher than those for PDT (*P* < .001). For multiple infections, the clearance rates were 64.0% (16/25) at 6 months and 84.0% (21/25) at 12 months, with no significant difference compared to PDT (*P* > .05).

These findings align with major studies: A Chinese multicenter, randomized, double-blind trial reported escalating Nr-CWS efficacy (67.2% → 93.4% over 12 months; *P* < .01) and superior HPV16/18 clearance (79.2%/73.3% vs controls; *P* < .001). The study also found that efficacy notably rose as the follow-up period extended after treatment (*P* < .01), with intermediate efficacy rates of 78.1% at 4 months and 89.1% at 8 months, each significantly outperforming the control group (*P* < .05).^[24]^ Another study observed a cumulative HR-HPV clearance rate of 79.13% following Nr-CWS treatment, with 163 out of 206 patients clearing the infection. Specifically, the treatment was effective in 73.13% of cases (49 out of 67) for single high-risk HR-HPV subtypes and in 71.43% of cases (20 out of 28) for multiple high-risk subtypes.^[25]^ Our 12-month outcomes (80% HPV16/18 clearance, 20 out of 25; 87.5% overall HR-HPV clearance, 63 out of 72) were consistent with these reports.

Limitations include the single-center retrospective design with limited sample size, potentially reducing statistical power and research value. Despite adjustment for 12 covariates via PSM, possible residual confounding factors could have influenced the allocation of patients to different groups.

## 5. Conclusion

The observed disparities in HR-HPV remission rates between Nr-CWS and PDT suggest superior efficacy of Nr-CWS in facilitating viral clearance. These findings highlight distinct advantages for each modality and underscore the importance of tailored approaches for HR-HPV management. For patients concerned about PDT-related side effects (e.g., pain, excessive discharge), Nr-CWS may be preferred—particularly in non-HPV16/18 monoinfections where it demonstrates higher clearance rates than PDT. For HPV16/18-positive patients or those with multiple infections who prioritize lesion clearance and tolerate PDT side effects, both treatments show comparable efficacy, with no statistically significant difference in clearance and cure rates. This study employed rigorous PSM to minimize confounding factors, providing robust evidence for noninvasive treatment selection in HR-HPV infection.

## Author contributions

**Conceptualization**: Suzhen Jiang, Hongxia Gong.

**Data curation**: Suzhen Jiang, Xiaomei Nan, Miaoxia Huang.

**Funding acquisition**: Suzhen Jiang.

**Formal analysis**: Jincheng Huang.

**Investigation**: Jincheng Huang.

**Methodology**: Jincheng Huang, Hongxia Gong.

**Resources**: Jincheng Huang, Xiaomei Nan, Miaoxia Huang.

**Project administration**: Xiaomei Nan, Miaoxia Huang, Hongxia Gong.

**Software**: Xiaomei Nan.

**Supervision**: Hongxia Gong.

**Writing – original draft**: Suzhen Jiang.

**Writing – review & editing**: Suzhen Jiang, Miaoxia Huang.
